# PREPARATION FOR FUTURE IN OLD AGE: WHY AND WHY NOT?

**DOI:** 10.1093/geroni/igz038.2748

**Published:** 2019-11-08

**Authors:** Helene H Fung

**Affiliations:** Chinese University of Hong Kong, Hong Kong, Hong Kong

## Abstract

With population aging, many people can expect to spend 30 or more years in old age. The five papers included in this symposium aim at shedding light on whether and how to make plans for old age, using data from the “Aging as Future” Project. First, Park and Hess used data spanning across adulthood from Germany, Hong Kong and the USA to examine how changes experienced in domains of functioning and the importance attached to these domains influenced preparations for old age. Next, de Paula Couto and Rothermund, examining Germans aged 40-90 years, pointed out that prescriptive age stereotypes might be the main drive for why people make preparations for age-related changes. The remaining three papers use qualitative data to qualify the above quantitative findings. Adamson and Ekerdt interviewed older Midwest US residents. They observed that SES greatly impacted how older adults perceived and made plans for their future. The final two papers examined how rural vs. urban contexts might affect preparations for future. Liou interviewed older adults in rural Tainan and found that their ideal old age was one about no future preparation, at least not about making plans for themselves (called “tranquil life”). Ho and colleagues, in contrast, found that for older Chinese residing in urban Hong Kong, not preparing for the future (called “time freeze”) was negatively related to physical and psychological well-being. The symposium will end with an overall discussion on future research directions on whether and how to make plans for old age.

